# Downstream optimization of fungal-based simultaneous saccharification and fermentation relevant to lignocellulosic ethanol production

**DOI:** 10.1186/s40064-015-0825-x

**Published:** 2015-02-01

**Authors:** Jin Seop Bak

**Affiliations:** Department of Chemical and Biomolecular Engineering, KAIST, 291 Daehak-ro, Yuseong-gu, Daejeon, 305-701 Republic of Korea

**Keywords:** Bioethanol, Electron beam irradiation, Fungal-based simultaneous saccharification and fermentation, Lignocellulose, *Mucor indicus*

## Abstract

To support the inefficient limitation of long-term biosystem by well-known simultaneous saccharification and fermentation (SSF), electron beam irradiated rice straw (at 80 kGy, 1 MeV, and 0.12 mA) was fermented using fungal-based simultaneous saccharification and fermentation (FBSSF) by saprophytic zygomycetes *Mucor indicus*. Based on the growth optimization (by response surface methodology), this eco-friendly bioprocess either without metabolic inhibitors (especially furfurals and acetic acids) or byproducts (especially glycerols) significantly increased the biodegradability and fermentability of lignocellulosic rice straw. Specifically, when irradiated straw was simultaneously bioconverted by *M. indicus* for 48 h, the ethanol yield was 57.2% of the theoretical maximum. This value was on the similar level as the 59.8% (for 144 h) measured from processed straw by well-known SSF. Furthermore, after FBSSF for 144 h based on large-scale mass balance, the ethanol concentration and production yield, and productivity were 34.6 g/L, 72.3% of the theoretical maximum, and 0.24 g/L/h, respectively.

**Electronic supplementary material:**

The online version of this article (doi:10.1186/s40064-015-0825-x) contains supplementary material, which is available to authorized users.

## Introduction

Forest bioenergy from renewable biomass has been actively reviewed as an alternative solution to the energy crisis (i.e., dependence on fossil fuels) and environmental instability (especially global warming) (Hudiburg et al. 2011). The stable production of cellulosic ethanol is dependent upon the extracellular bioconversion of crystalline-based fibers into biodegradable (or fermentable) monomers. Therefore, a few appropriate pretreatment processes (e.g., physicochemical and biochemical approach) is essential to increase the bioaccessibility (or porosity) of recalcitrant lignocellulosic substrates (Sanderson 2011; Chen and Dixon 2007). Recent trends of pretreatment technology have been focused on environmentally friendly programs (without inhibitory byproducts; e.g., furfurals) instead of traditional conventional processes using physicochemical methods (Menon and Rao 2012). Especially, electron beam irradiation (EBI)-based hydrolysis has been successfully developed for pretreatment of lignocellulosic feedstock (Bak et al. 2009; Hamm and Hamm 2012). In spite of high reaction rates based on enzymatic digestibility and structural crystallinity, this pretreatment method does not need the accessory processes (e.g., neutralization and cooling step).

Regarding the industrial utilization of cellulose fractions (i.e., released fermentable monomers), simultaneous saccharification and fermentation (SSF) process must be sequentially applied to biomass conversion program. However, based on time/cost effectiveness and metabolic stability, conventional SSF system (despite conventional approach by thermotolerant yeasts) may suffer from incomplete bioconversion, which is different reaction temperature between biodegradable enzymes and fermentable organisms (Kádár et al. 2004; Spindler et al. 1988). Recently, the fermentability of saprophytic zygomycetes has been extensively studied due to the metabolic balance (especially thermotolerant metabolism) and productivity (Ferreira et al. 2013). Particularly, in the presence of lignin (or cellulose or hemicellulose) derivatives, fermentation process with saprophytic *Mucor indicus* (formerly *M. rouxii*) has some advantages (e.g., lignocellulose utilization and thermotolerant growth) compared to that of *Saccharomyces cerevisiae* (Millati et al. 2005). However, the use of unsystemized fungal-based fermentation to enhance the fermentation yield of lignocellulosic substrates has not been sufficient for commercial programs yet. More importantly, it is hard for useful programs to maintain the spontaneous stability due to the inevitable necessity of long-term cultivation as well as substrate pretreatment.

Therefore, to address the limitations in the original SSF system, such as the low efficiency and the long-term cultivation, an EBI-treated substrate was used in optimized fungal-based simultaneous saccharification and fermentation (FBSSF) program. This study was conducted to verify the industrial feasibility and efficiency of advanced FBSSF program. Its impact was evaluated based on various downstream indexes of pretreated substrate, such as biodegradability yield and fermentation capacity.

## Materials and methods

### Strain and cultivation conditions

*Mucor indicus* ATCC 24905 was used in this study. The spores taken from cultures grown on potato dextrose agar plates were inoculated at 2.1 × 10^6^ spores/mL, and then incubated at 28°C with shaking at 200 rpm for 72 h. The spore concentration was checked by suspending conidia in 0.85% (w/v) sterile saline and then counting the spores in a cell counting chamber (Neubauer**,** Marienfeld**,** Germany).

### Fungal-based simultaneous saccharification and fermentation

After the minimal preprocessing (e.g., washing, air-drying, and milling; Additional file 1), lignocellulosic rice straw (RS) was used as the starter material for the fungal-based simultaneous saccharification and fermentation (FBSSF). Prior to the fermentation, RS was pretreated by using an electron-beam linear accelerator (Korea Atomic Energy Research Institute, Daejeon, Korea) in order to induce the disruption of recalcitrant materials. The stable condition (1 Mev and 80 kGy at 0.12 mA) of irradiation pretreatment was based on a previously confirmed downstream efficiency for lignocellulosic hydrolysis (Bak et al. 2009b). Next, based on the National Renewable Energy Laboratory (NREL) public protocol with slight modification (http://www.nrel.gov/biomass/) (Bak et al. 2009a), advanced FBSSF using RS substrates with a glucan of 3.1% (w/v) in 250 mL of statistically optimized medium (for cell population) was performed using *M. indicus* as well as 15 FPU of cellulase (Celluclast 1.5 L, Sigma-Aldrich, St. Louis, MO) and 30 CBU of β-glucosidase (Novozyme 188, Sigma-Aldrich) per gram of glucan at an initial pH of 5.0. The samples were cultured at 38°C and 150 rpm for 144 h. Additionally, Avicel (Sigma-Aldrich) and untreated RS were also used in the fermentation test as control substrates. In particular, *Saccharomyces cerevisiae* D5A (ATCC 200062) was used as fermentable organism for traditional simultaneous saccharification and fermentation process.

### Statistics-based optimization for fermentation process

In order to control various parameters (especially growth rate and pH) in fungal biosystem, based on generally accepted procedures via response surface methodology (RSM)-based statistics (especially Plackett-Burman design [PBD] and central composite design [CCD]; Myers and Montgomery 1995), process optimization for cell population was carried out. Further details are provided in Additional file 1. Finally, the top 3 components for optimized fermentation were determined as yeast extract, KH_2_PO_4_ and glucose, and these components were subsequently optimized by central composite methodology. For the significant validation of RSM model, analysis of variance (ANOVA) and platform of design matrices were carried out using the SAS 9.2 (SAS Institute, Cary, NC) and SigmaStat 3.5 (Systat Software, San Jose, CA).

### Downstream data analysis and industrial evaluation

The production of metabolic byproducts (especially HMF, furfural, acetate, cellobioase, and glycerol) and theoretical yields (especially biodegradability and fermentability) in the FBSSF-treated biosystem were analyzed following the NREL protocols. Further details are provided in Additional file 1. The degree of simultaneous fermentability (Eq. 1) was indicated as a percentage of the theoretical maximum of biodegradable substrates obtained from untreated RS material. All experiments were conducted in triplicate.

1 $$ \mathrm{Ethanol}\ \mathrm{yield}\ \left(\%\ \mathrm{theoretical}\ \mathrm{maximum}\right)\kern0.37em =\kern0.62em \frac{\mathrm{g}\ \mathrm{of}\ \mathrm{ethanol}}{\mathrm{g}\ \mathrm{of}\ \mathrm{glucan} \times 1.1\times 0.511}\times 100 $$

## Results and discussion

### Growth optimization of *M. indicus* for biomass fermentation

In order to improve the efficiency of FBSSF program (by *M. indicus*), the productivity of fungal spore was statistically maximized based on medium optimization (especially by PBD and CCD approach). In preliminary experiments (Additional file 1: Tables S1 and S2), yeast extract and glucose were found to inducing significantly higher populations of the spore compared to other nitrogen and carbon sources, respectively. Based on the predominant targets, the effects of other medium components on the spore productivity were also evaluated using a 7 × 8 PBD (Table 1 and Additional file 1: Table S3). As a result, the order of the impact of each component was FeSO_4_ > yeast extract > KH_2_PO_4_ > glucose > MgCl_2_ > vitamin solution > CaCl_2_. Especially, FeSO_4_ were found to greatly affect the volumetric productivity of *M. indicus* as seen in the PBD analysis. In the presence of lignocellulosic polysaccharides, although ferric/ferrous network (from FeSO_4_) may be a cofactor in Fenton cascades that supplies reactive oxygen species-based radicals for the peroxidative modification (i.e., exposure of fermentable sugars) of recalcitrant lignins (Bak 2015; Bak 2014a), in this study, cell growth was inhibited by an increase (i.e., above 0.1 g/L) in FeSO_4_. Therefore, the first ranked FeSO_4_ were added to media at the final concentration of 0.01 g/L, but they were not considered in the CCD analysis for further medium optimization.

**Table 1:** **Analysis of multiple variables based on the PBD methodology in FBSSF process by** ***M. indicus***

**Run**	**Elements**							**Spore production(lnY)**
	**Glucose(g/L)**	**Yeast extract(g/L)**	**KH**_**2**_ **PO**_**4**_ **(g/L)**	**CaCl**_**2**_ **(g/L)**	**MgCl**_**2**_ **(g/L)**	**FeSO**_**4**_ **(g/L)**	**Vitamin Solution(ml/L)**	
1	25	12.5	0.94	0.0236	3.267	2.388	29.25	17.7339 ± 0.4175
2	25	4.17	0.94	0.0097	9.44	9.722	4.875	17.8051 ± 0.1877
3	25	4.17	3.33	0.0097	3.267	2.388	4.875	19.4832 ± 0.0711
4	8.33	12.5	0.94	0.0097	3.267	9.722	4.875	16.9431 ± 0.3296
5	8.33	12.5	3.33	0.0236	9.44	2.388	4.875	18.8097 ± 0.5846
6	8.33	4.17	3.33	0.0236	3.267	9.722	29.25	17.8758 ± 0.2263
7	8.33	4.17	0.94	0.0097	9.44	2.388	29.25	19.6368 ± 0.1854
8	25	12.5	3.33	0.0097	9.44	9.722	29.25	17.3475 ± 0.3142

Based on the canonical analysis after the 3 × 16 CCD analysis (Tables 2 and 3), a 2^nd^-order polynomial expression was calculated as Eq. 2. Under these optimal conditions, the predicted yield (lnY) of spore production was 22.0287. To check the validity of the predicted values, the experiments were performed at the above optimal conditions. The actual yield (lnY) from the large-scale fermentations was 21.42 ± 0.25, and the predicted values were in good agreement with the experimental values. To validate the model equation and the statistical results, ANOVA was carried out as shown in Table 4. The results revealed that the yield of the spores produced differed significantly among groups (*P* < 0.05). In addition, the model obtained from the RSM had an *R*^2^ of 0.9246, which indicated that 92.46% of the total variation could be accounted for by the equation.

**Table 2:** **Basic CCD analysis of the predominant target variables in FBSSF by** ***M. indicus***

**Run**	**Level**			**Spore production(lnY)**
	**Glucose(g/L)**	**Yeast extract(g/L)**	**KH**_**2**_ **PO**_**4**_ **(g/L)**	
1	7.5	3	1.5	21.34182
2	7.5	3	4.5	21.72573
3	7.5	5	1.5	21.59873
4	7.5	5	4.5	21.56699
5	12.5	3	1.5	21.17752
6	12.5	3	4.5	21.51739
7	12.5	5	1.5	21.02337
8	12.5	5	4.5	21.55073
9	10	4	3	22.20487
10	10	4	3	21.89420
11	6.79	4	3	21.52302
12	13.21	4	3	21.29000
13	10	2.72	3	21.17752
14	10	5.28	3	22.02482
15	10	4	1.074	21.95437
16	10	4	4.926	21.75289

**Table 3:** **Canonical analysis of response surface points based on coded data in optimized FBSSF biosystem by** ***M. indicus***

**Code**	**Glucose (g/L)**		**Yeast extract (g/L)**		**KH**_**2**_ **PO**_**4**_ **(g/L)**	
	**x**_**1**_	**X**_**1**_	***x***_**2**_	***X***_**2**_	**x**_**3**_	**X**_**3**_
Critical value	- 0.08088	9.74036	0.14512	4.18576	0.61716	4.18865

X_1_=3.2x_1_+10; *X*_2_=1.3x_2_+4; X_3_=0.3x_3_+4.

**Table 4:** **Factorial ANOVA analysis for the optimization of spore population in optimized FBSSF by** ***M. indicus***

**Source**	**Sum of squares**	**Degree of freedom**	**Mean square**	***F*** **-value**	***P*** **>** ***F***	***R*** ^***2***^
Model	1.52199	10	0.15219	5.46	0.0374	0.9161
Error	0.13939	5	0.02787			
Corrected total	1.66139	15				

2 $$ \begin{array}{l}\mathrm{y}=13.2645+0.9628{\displaystyle {X}_1}+1.8628{\displaystyle {X}_2}+0.0839{\displaystyle {X}_3}-0.0109{\displaystyle {X}_2}{\displaystyle {X}_1}+0.0171{\displaystyle {X}_3}{\displaystyle {X}_1}-\\ {}0.0190{\displaystyle {X}_3}{\displaystyle {X}_2}-0.0507{\displaystyle {X}_1^2}-0.2002{\displaystyle {X}_2^2}-0.0204{\displaystyle {X}_3^2}\end{array} $$

**Figure 1:**
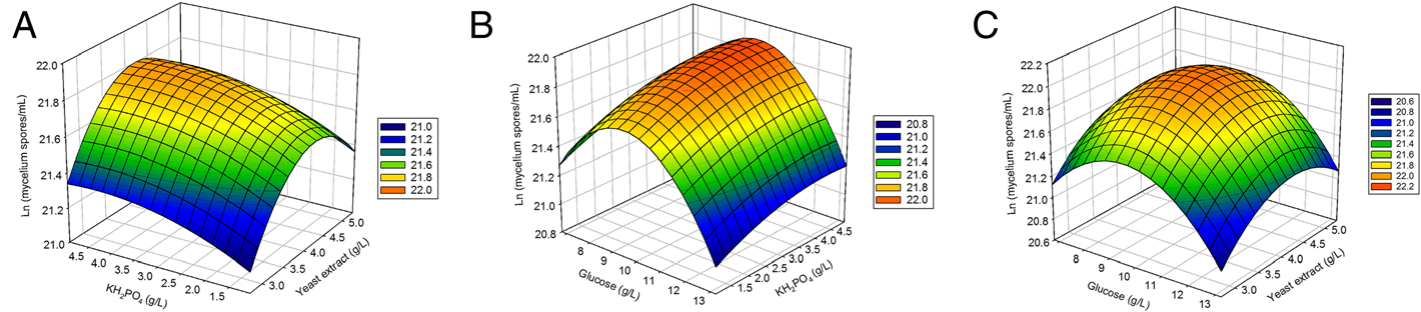
**Response surface plots of the interaction between three selected variables and the spore productivity of** ***M. indicus*** **in optimized FBSSF process.** **(A)** Yeast extract and KH_2_PO_4_ with a fixed concentration of glucose. **(B)** KH_2_PO_4_ and glucose with a fixed concentration of yeast extract. **(C)** Yeast extract and glucose with a fixed concentration of KH_2_PO_4_. Additional nutrient was 0.001% (w/v) FeSO_4_ for each of the experiments.

The response surface plots of yeast extract, KH_2_PO_4_, and glucose (Figure 1) revealed that the spore productivity was strongly dependent on the three elements. In particular, the productivity showed distinct optimal points depending on the concentration of KH_2_PO_4_ when glucose or yeast extract was fixed at their optimal values as shown in Figure 1B or 1C, respectively. When KH_2_PO_4_ was fixed at its optimal value, the productivity generally increased with an increase in yeast extract or glucose as shown in Figure 1A.

### Industrial maximum yields of FBSSF system

In order to evaluate the fermentability of optimized FBSSF process, the EBI-irradiated RS was simultaneously biodegraded by the addition of both cellulase and β-glucosidase. Ethanol indexes (after 48 h; nearly stationary phase based on industrial process) were 57.2% and 47.2% from treated RS with bioconversion types of FBSSF and original SSF (by *S. cerevisiae* D5A), respectively (Table 5). Especially, when original SSF was treated, the maximal yield was determined to be 59.8% in spite of long-term fermentation of 144 h. Based on the biodegradability of RS substrate, probably, this phenomenon (under the original SSF) may have been shown due to the higher uptake of glucose by fermentable yeast *S. cerevisiae* (rather than the release of glucose from the substrate). On the other hand, because the fermentation by *M. indicus* has a competitive advantage based on the utilization of hemicellulosic monomers (e.g., arabinose and xylose) and oligomers (e.g., cellobiose and maltose) (Millati et al. 2005; Ueng and Gong 1982), the biodegradability in optimized large-scale FBSSF biosystem is relatively stable (Additional file 1: Table S1). For reference, regarding the similar pattern (below 2.2%; < 0.7 g glucose/L; Table 5) of maximum yields, it implies that intracellular stability have significant effect on fermentation efficiency, which predicts at proactive compensatory metabolisms (e.g., secondary metabolisms; especially glycerol; Table 6) to keep metabolic homeostasis, regardless of either substrate pretreatment or cell type. As the effect of EBI process has shown, the fermentability of untreated RS was just 25.8% of the maximum ethanol yield regardless of long-term cultivation (over 144 h). Probably, the modified repositioning of polymeric structures (especially crystalline polymers) by peroxidative radicals from the EBI may accelerate to the access of fermentation-mediated enzymes (Bak 2014d), and thus can shorten a lengthy time of fermentation process for % yield maximum. Interestingly, the fermentability (after 144 h; stationary phase) from the FBSSF system was approximately 2.8 times higher than that of untreated sample, which is likely due to the proactive formation of cellulolytic platforms based on the open structure of pretreated polymers.

**Table 5:** **Downstream index for scale-up in advanced FBSSF program**

**Type**	**External substrate**	**Evaluation parameter for industrial applications**		
		**Biodegradability** ^**d**^	**Fermentability** ^**e**^	
			**Fermentation for 48 h**	**Fermentation for 144 h**
FBSSF	33.8 g RS^c^	≤9.3% (3.0 ± 0.1 g glucose/L)	≤57.2% (9.3 ± 0.2 g ethanol/L)	≤72.3% (11.7 ± 0.2 g ethanol/L)
SSF^a^	33.8 g RS^c^	≤8.3% (2.7 ± 0.1 g glucose/L)	≤47.2% (7.7 ± 0.2 g ethanol/L)	≤59.8% (9.7 ± 0.2 g ethanol/L)
Untreated^b^	34.5 g RS	≤7.1% (2.3 ± 0.1 g glucose/L)	≤22.7% (3.7 ± 0.1 g ethanol/L)	≤25.8% (4.2 ± 0.1 g ethanol/L)
Untreated^b^	12.4 g Avicel	≤11.0% (3.5 ± 0.1 g glucose/L)	≤74.4% (12.0 ± 0.2 g ethanol/L)	≤82.4% (13.3 ± 0.2 g ethanol/L)

^a^SSF by *S. cerevisiae* D5A (ATCC 200062); Bak 2014d.

^b^FBSSF without the EBI.

^c^loss (approximately 2%) of RS substrate by the EBI.

^d^the maximum yield of soluble monomeric sugar after 24 h of fermentation.

^e^the yield of theoretical maximum ethanol.

**Table 6:** **Analysis of lignocellulose-based biorefinery byproducts in advanced FBSSF**

**Type**	**Cellulose-based dimer** ^**c**^ **(g cellobiose/L)**	**Glycerol (g/L)**	**Target metabolic inhibitors**		
			**Furfural** ^**d**^ **(w/w, %)**	**HMF** ^**d**^ **(w/w, %)**	**Acetate (g/L)**
FBSSF	<0.3	<2.0	Not detected	Not detected	<0.1
SSF^a^	<0.2	<1.8	Not detected	Not detected	<0.1
Untreated^b^ (RS)	<0.2	<0.5	Not detected	Not detected	<0.04
Untreated^b^ (Avicel)	<0.4	<3.0	Not detected	Not detected	<0.1

^a^SSF by *S. cerevisiae* D5A (ATCC 200062); Bak 2014c.

^b^FBSSF without the EBI.

^c^glucose dimer from the biodegradable substrates after 96 h of fermentation.

^d^determined as $\frac{\mathrm{g}\ \mathrm{furfural}\ \mathrm{of}\ \mathrm{treated}\kern0.5em \mathrm{R}\mathrm{S}}{\mathrm{g}\ \mathrm{of}\ \mathrm{initial}\ \mathrm{weight}\ \mathrm{of}\ \mathrm{R}\mathrm{S}}\times 100$ and $\frac{\mathrm{g}\ \mathrm{of}\ \mathrm{H}\mathrm{M}\mathrm{F}\ \mathrm{of}\ \mathrm{treated}\kern0.5em \mathrm{R}\mathrm{S}}{\mathrm{g}\ \mathrm{of}\ \mathrm{initial}\ \mathrm{weight}\ \mathrm{of}\ \mathrm{R}\mathrm{S}}\times 100$.

Overall, the fermentation yield (72.3%; Table 5) of advanced FBSSF program, which is reflected in biomass-based ethanol, was higher than those (67.6%) of lignocellulosic feedstock pretreated using conventional chemical program (especially by dilute acids; Karimi et al. 2006). However, the ethanol productivity (0.25 g/L/h after 48 h) of conventional platform (especially optimized ammonia-soaking based on original SSF) (Ko et al. 2009) are greater than the productivity (0.19 g/L/h) observed in the present study. Interestingly, the fermentability of microbial-based biosystem was finally obtained as below 0.03 g bioethanol/g biomass after 144 h of SSF (Shi et al. 2009; Shrestha et al. 2008), which was not more than that of the FBSSF biosystem (0.08 g ethanol/g RS).

### Substrate-mediated metabolic compounds and byproducts

Regarding the generation of inhibitory byproducts against either the metabolic bioconversion or fermentation, although the theoretical indexes of the FBSSF-treated straw were not higher than those of lignocellulose pretreated using conventional approaches, the activation of inhibitors, such as acetates, HMFs, and furfurals, was either negligible or not detected (Table 6). Especially, the activation of byproducts from the physicochemical treatment (especially dilute acid program; Merino and Cherry 2007) was found to result in higher than that of the green biochemistry-based platform (here EBI-based program). Regarding the metabolic cascades in fermentation biosystem, the intentional induction of major metabolic byproducts (especially cellobioses and glycerols; Table 6) was also found to result in higher intracellular bioconversion compared with substrate consumption (% theoretical indexes; Table 5) on byproduct accumulation.

### Scale-up mass balance and economic feasibility

Based on the same assumption for untreated groups (Table 5), when 100 g of initial RS was consecutively subjected to EBI pretreatment and then optimized scale-up FBSSF, 34.6 g (here untreated RS, 12.1 g; Avicel, 38.5 g) of ethanol was produced after 144 h. Additionally, the ethanol yield from irradiated rice straw in traditional SSF was 59.8% (i.e., 28.7 g ethanol; here untreated RS, 23.4%) of the theoretical maximum.

In a practical aspect of open bioconversion platform, when compared to conventional pretreatment programs (e.g., dilute-acid and ammonia-soaking), the irradiation-based FBSSF process may be regarded inefficient based on the initial running costs (especially high-power consumption and related equipment) of the EBI linear accelerator. However, interestingly, the accelerator is considerably useful in case of large-scale process (over 100 g biomass) owing to the advantage of one-step platform without an orderly repetition of individual preparation process (i.e., preheating/stabilizing step). More remarkably, unlike the conventional programs, the EBI-based biosystem does not need additional costs (especially neutralization and heating/cooling process) after the main bioconversion processes (especially biomass hydrolysis and fermentation) (Bak 2014c). Furthermore, the most important issue for this biosystem is the effectiveness of total process time (approximately 5 min except for the preparation time).

## Conclusions

In order to upgrade the theoretical efficiency of either traditional SSF or fungal-based fermentation, RS was pretreated to improve the biodegradation accessibility by using an optimized EBI at 0.12 mA-80 kGy-1 MeV. Based on the optimized FBSSF program (by statistical PBD and CCD), pretreated RS showed increases in the biodegradability (9.3% of the theoretical maximum) of crystalline substrates as well as in ethanol production (72.3% of the theoretical maximum) in FBSSF, compared with those of the untreated RS. Particularly, the reduction of a lengthy time in advanced FBSSF-program (compared with the traditional SSF) had a strong advantage in stable industrial downstream. Although the yields of the advanced program was lower than those of substrate pretreated by physicochemical systems, the inhibitory compounds was rarely generated. Lastly, the approach secured a bridgehead for energy/time saving in conventional competitiveness. However, no "conventional programs" (i.e., benchmark treatment runs) were examined in the present study.

## Additional file

Additional file 1: **Supplementary Materials and Methods.** **Table S1.** Spore production of *M. indicus* in FBSSF process with various carbon sources. **Table S2.** Spore production of *M. indicus* by FBSSF in the presence of various organic and inorganic nitrogen sources. **Table S3.** Ranking of significant multiple variables for the CCD based on the PBD in FBSSF by *M. indicus*.
