# Supplementary material for: Downstream optimization of fungal-based simultaneous saccharification and fermentation relevant to lignocellulosic ethanol production
Source: Springerplus. 2015 Feb 1;4:47. doi: 10.1186/s40064-015-0825-x (PMC4334921; doi:10.1186/s40064-015-0825-x)
Supplement: Supplementary file 1 — Supplementary Materials and Methods. Table S1. Spore production of M. indicus in FBSSF process with various carbon sources. Table S2. Spore production of M. indicus by FBSSF in the presence of various organic and inorganic nitrogen sources. Table S3. Ranking of significant multiple variables for the CCD based on the PBD in FBSSF by M. indicus. [file 40064_2015_825_MOESM1_ESM.doc]

**Supporting Information**

Downstream optimization of fungal-based simultaneous saccharification and fermentation relevant to lignocellulosic ethanol production

Jin Seop Bak

*Department of Chemical and Biomolecular Engineering, Advanced Biomass R&D Center, KAIST, 291 Daehak-ro, Yuseong-gu, Daejeon, 305-701, Republic of Korea*

*Address correspondence to J. S. Bak, jsbwvav7@kaist.ac.kr.*

**Supplementary Meterials and methods**

**Preperation of lignocellulosic substrate**

Air-dried RS at room temperature was harvested from Korea University Farm (Deokso, Korea). The RS was then processed using a cutting mill (MF 10, IKA, Staufen, Germany), after which it was sieved using 425 and 710 μm sieves. Next, they were dried in a vacuum-drying oven at 45 ^o^C for 5 days one more time until 96.6% (w/w) of solid content. Prior to the fungal fermentation, treated RS was autoclaved at 121°C for 10 min. Based on the NREL protocols, the compositional analysis revealed that the untreated RS consisted of 36.0% glucan, 3.7% mannan, 2.5% galactan, 10.8% xylan, 3.3% arabinan, and 19.7% lignin on a dry weight basis.

**RSM-based optimization of fungal medium for cell growth**

Similar to well-known RSM-related procedures (ref. 1), a dependency of carbon sources (1%, w/v) including arabinose, cellobiose, cellulose, glucose, galactose, maltose, xylose, sucrose, and starch were evaluated in the media containing 0.5% (w/v) of yeast extract. Furthermore, the effect of various nitrogen sources (0.5%, w/v) including (NH_4_)_2_SO_4_, yeast extract, peptone, glycine, and urea were checked in media containing 1% of glucose. Based on the preliminary tests, medium containing both glucose and yeast extract was then optimized using Plackett-Burman protocol (ref. 2) with other substrates such as vitamin solution, KH_2_PO_4_, CaCl_2_, MgCl_2_, and FeSO_4_. In addition, the vitamin stock consisted of the following components (all from Sigma-Aldrich, St. Louis, MO): *myo*-inositol, 25 g/L; nicotinic acid, 1 g/L; (+)-pantothenic acid, 1 g/L; calcium salt hydrate, 1 g/L; pyridoxine hydrochloride, 1 g/L; thiamine hydrochloride, 1 g/L; 4-aminobenzoic acid, 0.2 g/L; and biotin, 0.05 g/L. For reference, after the central composite design analysis (ref. 1), ridge analysis (for out of range) did not applied to predict a locus of the response surface point within central composite range.

**Analysis of downstream metabolites**

For biodegradability analysis (i.e., monomeric sugars), HPLC (Agilent 1100, Agilent Technologies, Waldbronn, Germany) equipped with a RID (Agilent Technologies) and a Shodex SP-0810 column (Pb^2+^ form; Showa Denko, Tokyo, Japan) was used in the present study. Extracellular metabolic byproducts (especially acetates and glycerols) were quantified by HPLC equipped with a RID and an Aminex HPX-87H column (Bio-Rad, Richmond, CA). The mobile phase, 0.01 N H_2_SO_4_, was applied at a flow rate of 0.6 mL/min at 55°C.

**Supplementary** **table legends**

**Table S1.** Spore production of *M. indicus* in FBSSF process with various carbon sources.

**Table S2.** Spore production of *M. indicus* by FBSSF in the presence of various organic and inorganic nitrogen sources.

**Table S3.** Ranking of significant multiple variables for the CCD based on the PBD in FBSSF by *M. indicus*.

**Supplementary References**

1. Myers RH, Montgomery DC. 1995. Response surface methodology: process and product optimization using designed experiments. New York: John Wiley & Sons, Inc.
2. Plackett RL, Burman JP. 1946. The design of optimum multifactorial experiments. Biometrika 33:305-325.

**Table S1.** Spore production of *M. indicus* in FBSSF process with various carbon sources.

| Type | Substrate | Concentration  (%, w/v) | Spore production (×10^8^) |
| --- | --- | --- | --- |
| Control ^a^ | - | - | 0.016 ± 0.006 |
| Monosacchride (Hexose)  Monosacchride (Hexose) | glucose  galactose | 1.0  1.0 | 12.40 ± 0.60  11.20 ± 0.30 |
| Monosacchride (Pentose)  Monosacchride (Pentose) | xylose  arabinose | 1.0  1.0 | 5.10 ± 0.40  3.67 ± 0.50 |
| Oligosacchride  Oligosacchride  Oligosacchride | maltose  sucrose  cellobiose | 1.0  1.0  1.0 | 10.10 ± 0.20  0.60 ± 0.30  3.50 ± 0.10 |
| Polysacchride (crystalline complex)  Polysacchride | cellulose  starch | 1.0  1.0 | 0.10 ± 0.10  4.77 ± 0.80 |

Nitrogen source: 0.5% (w/v) yeast extract.

^a^ No substrate was added to the control culture.

**Table S2.** Spore production of *M. indicus* by FBSSF in the presence of various organic and inorganic nitrogen sources.

| Type | Substrate | Concentration  (%, w/v) | Spore production  (×10^8^) |
| --- | --- | --- | --- |
| Control ^a^  Organic source  Organic source  Organic source | -  Yeast extract  Peptone  Glycine | -  0.5  0.5  0.5 | 0.016 ± 0.006  12.40 ± 0.60  0.10 ± 0.10  0.20 ± 0.20 |
| Inorganic source  Inorganic source | Urea  (NH_4_)_2_SO_4_ | 0.5  0.5 | 0.10 ± 0.10  0.13 ± 0.10 |

Carbon source: 1.0% (w/v) glucose.

^a^ No substrate was added to the control culture.

**Table S3.** Ranking of significant multiple variables for the CCD based on the PBD in FBSSF by *M. indicus*.

| Variable element | E(x,i) ^a^ | |E(x,i)| | Ranking |
| --- | --- | --- | --- |
| Glucose | - 0.1661 | 0.1661 | 4 |
| Yeast extract | - 0.3975 | 0.3975 | 2 |
| KH_2_PO_4_ | - 0.2046 | 0.2046 | 3 |
| CaCl_2_ | - 0.0229 | 0.0229 | 7 |
| MgCl_2_ | - 0.1252 | 0.1252 | 5 |
| FeSO_4_ | - 0.6436 | 0.6436 | 1 |
| Vitamin Solution | - 0.0867 | 0.0867 | 6 |

^a^ E(x,i) = (∑X_i+_ – X_i-_) / Number of experiments.
